# Feasibility and acceptability of a video library tool to support community health worker counseling in rural Afghan districts: a cross-sectional assessment

**DOI:** 10.1186/s13031-020-00302-z

**Published:** 2020-08-05

**Authors:** Leila C. Dal Santo, Sayed Haroon Rastagar, Shafiqullah Hemat, Sayed Omar Alami, Subarna Pradhan, Jenae Tharaldson, Lisa S. Dulli, Catherine S. Todd

**Affiliations:** 1 Global Health, Population, & Nutrition Division, FHI 360, Durham, North Carolina USA; 2 Boston College School of Social Work, 140 Commonwealth Avenue, Chestnut Hill, Massachusetts 02467 USA; 3 FHI 360/ HEMAYAT project, Kabul, Afghanistan; 4 Health Promotions Department, Ministry of Public Health, Islamic Republic of Afghanistan, Kabul, Afghanistan

**Keywords:** Afghanistan, Community health worker, Maternal and child health, Counseling, Social and behavior change, Fragile states

## Abstract

**Background:**

Rural Afghan populations have low skilled birth attendance rates and high maternal and infant mortality. Insecurity and armed conflict, geographic barriers, and cultural norms often hinder women’s access to facility-based reproductive, maternal, newborn, and child health (RMNCH) services. Community health workers (CHWs) are critical agents for behavioral change in this and similarly fragile settings, where RMNCH information exposure is limited by low literacy and mass media access. We assessed the feasibility and acceptability of a computer tablet-based health video library (HVL) to enhance CHW counseling on RMNCH topics in three rural Afghan districts.

**Methods:**

The HVL was introduced by trained CHWs in 10 pilot communities within one rural district in each of Balkh, Herat, and Kandahar provinces. We used a mixed-methods study design to assess exposure to and perception of the HVL 6 months post-introduction. We surveyed married women (*n* = 473) and men (*n* = 468) with at least one child under 5 years and conducted in-depth interviews with CHWs and community leaders (*shuras* and Family Health Action groups) within pilot communities (*n* = 80). Program improvement needs were summarized using quantitative and qualitative data.

**Results:**

Higher proportions of women in Balkh (60.3%) and Herat (67.3%) reported viewing at least one HVL video compared to women in Kandahar (15%), while male HVL exposure was low (8–17%) across all districts. Most HVL-exposed clients (85–93% of women and 74–92% of men) reported post-video counseling by CHWs. Nearly all (94–96% of women and 85–92% of men) were very interested in watching videos on other health topics in the future. Participants recommended increasing the number of videos and range of topics, using tablets with larger screens, and translating videos into additional local languages to improve the HVL program.

**Conclusion:**

The HVL was a highly acceptable tool for relaying health information, but coverage of female audiences in Kandahar and male audiences broadly was low. The HVL should better engage men and other key influencers to engineer local solutions that directly facilitate male HVL exposure, indirectly improve women’s HVL access, and support collaborative spousal health decision-making. A larger efficacy trial is warranted to measure the HVL’s effect on knowledge and health-related behavioral outcomes.

## Introduction

Afghanistan has experienced nearly four decades of uninterrupted armed conflict, accelerating in the last decade [1]. Ongoing political insecurity in this fragile state continues to impede development efforts, including those in the health sector. While reproductive, maternal, newborn and child health (RMNCH) in Afghanistan has steadily improved over the last 15 years [2], the country has among the highest maternal, infant, and child mortality rates regionally [3]. Contributing to these high mortality rates is the fact that rural Afghan populations have less access to facility-based health services and to health messages delivered by mass media or health providers [2, 4–6]. Insecurity and armed groups, geographic barriers, and cultural norms hinder direct access to facility-based services [7–10], while low exposure to RMNCH information in these settings results from low literacy and limited mass media access [2, 5, 6].

Within this context, community health workers (CHWs) are a critical channel for motivating relevant health behavior change, particularly for RMNCH [11, 12]. Volunteer CHWs, comprising one male and one female CHW for each health post (catchment area of up to 150 households), are trained and then charged to provide health promotion, family planning (FP), childhood illness treatment, and facility referrals to members of their communities [12, 13]. Due to gender norms in Afghanistan, female CHWs generally interact solely with female clients within households and health posts, while male CHWs convene education and mobilization sessions for male clients at community events and coordinate referrals [11, 13].

Mobile health (mHealth) modalities to support health promotion and care provision, like short message service (SMS) and videos, have gained traction in recent years in a range of settings [14–18]. These tools offer promise in contexts where inadequate training or staffing, multiple competing priorities, and lack of supplies and access to other communication channels often hamper the effectiveness of CHW-led RMNCH interventions [18–20]. While mHealth interventions in low- and middle-income countries are usually delivered via SMS and interactive voice response (IVR) technologies, visual media within mHealth are under-utilized for engaging clients, their families, and communities in RMNCH promotion [21, 22]. Interventions in sub-Saharan Africa and South Asia have used video content to enhance health provider counseling. In urban South Africa, a pilot program incorporated videos related to HIV, nutrition, and breastfeeding as part of CHW counseling during home visits. CHWs reported that these tools enabled them to better engage clients and to educate clients’ families and the community at large [21]. In rural India, mobile phone videos were used by nurse midwives to support patient education during postnatal care examinations [23] and by Accredited Social Health Activists (ASHAs) [24] to encourage pregnant women and their families to utilize health services. Nurse midwives reported increased patient trust and enhanced work authority [23], while ASHAs cited increased engagement with clients and their families [24]. However, there is little evidence regarding the feasibility and acceptability of videos as counseling aids among clients.

In Afghanistan, the United States Agency for International Development (USAID)-funded Helping Mothers and Children Thrive in Afghanistan (HEMAYAT) project aims to reduce maternal and child mortality in part by increasing RMNCH service demand within communities [25]. Inspired by the Family Health Book Program [26], in which audiovisual devices augmented household adult literacy and heath training, the health video library (HVL) activity was pilot-tested in three rural Afghan districts beginning in July, 2017 through a collaborative effort by the Ministry of Public Health (MoPH) and HEMAYAT. The HVL program uses short educational videos portraying specific health topics to support CHW counseling, with the aim of improving RMNCH knowledge and practices among community members. As part of this activity, the Health Promotions Department (HPD) of the MoPH and the HEMAYAT project jointly conducted an assessment to determine HVL feasibility and acceptability and to identify strategies to strengthen activity design prior to scale-up. This assessment evaluated operator perspectives on the feasibility and acceptability of the HVL tool and its utility in engaging clients; a separate manuscript describing these results is currently under review. In this manuscript, we present findings from the assessment that highlight the client perspective and provide recommendations to improve activity design and content prior to scale-up in focus provinces.

## Methods

### Intervention description

The HVL activity is a computer tablet-based video activity for CHWs to show health information videos to clients, followed by guided discussions and referrals to health services as needed. The HVL design was informed by a formative study among youth of both sexes and adult men conducted earlier in the HEMAYAT project (between March and July 2017), which found that visual communication channels were preferable to most target groups, particularly female youth [27]. HVL content was selected based on topics prioritized by the MoPH and gaps identified in this assessment [27]. Videos were developed in collaboration with the MoPH on topics for which there were not pre-existing MoPH-approved videos, such as postpartum hemorrhage (PPH) prevention, postpartum intrauterine contraceptive device (PPIUCD) use, respectful maternity care and gender issues in RMNCH care, lactational amenorrhea method (LAM) for FP, and routine newborn care. Videos were produced in Kabul in Dari and Pashto languages, the predominant national languages. These new videos as well as pre-existing MoPH-approved content on nutrition and immunization topics were loaded onto tablets, creating the “video library.” The teams also developed accompanying counseling materials containing key messages and action points for CHWs to convey after showing the videos. The newly created videos, training manual, and post-video counseling guidelines were pre-tested in four provinces with ethnic and linguistic diversity prior to implementation.

The VL pilot intervention comprised distributing tablets loaded with health video content to trained CHWs, who were then encouraged to show the videos to clients—either in individual homes or during group sessions in the communities where CHWs are based—and to provide structured counseling. The national CHW training package comprises 9 weeks of didactic training and 8 weeks of practical training which must be completed satisfactorily prior to certification. This training package includes counseling techniques, sensitization, and social norms accommodation to facilitate community engagement on sensitive topics, like family planning. The HVL pilot activity was implemented in 30 villages in Char Kent District (Balkh Province), Karukh District (Herat Province), and Daman District (Kandahar Province). The selected provinces are distinct from each other by geography and ethnic distribution, and the pilot districts are rural and predominantly agrarian. We purposefully selected diverse sites to determine whether activity design was broadly acceptable and to ensure that regional preferences were considered in implementation during scale-up. For each province, one district was selected randomly from secure, accessible rural districts with operational health posts staffed by established female CHWs. Based on input from the Provincial Health Directorate and the Basic Package of Health Services (BPHS) implementing organization, 10 eligible female CHWs residing in the selected district of each province were chosen and offered the opportunity to implement the HVL program. Female CHWs were selected based on the following criteria: known to be currently working at their health post within the last month who had access to and the ability to use a mobile phone, as identified through the Community Health Supervisor. If there were more than 10 female CHWs identified meeting eligibility criteria affiliated with the specific health facility, the CHS purposefully chose the CHWs with the highest known activity via health post sessions and home visits. If a CHW declined participation, another CHW with the same criteria was chosen to replace her to reach the planned sample size.

CHWs attended a four-day training inclusive of tablet use, video content and counseling techniques, and approaches to HVL activity promotion within their communities. The tablets were signed over to the custody of the female CHWs as the primary implementer. Implementation began immediately following the training, with field-based assistance from the HEMAYAT team and MoPH representatives during the first week to ensure CHWs competently operated the tablet and provided post-video counseling. Specifically, HEMAYAT staff observed each CHW conduct at least one HVL session (during which up to three videos were shown) at a health post or similar facility and provided coaching prior to wider HVL promotion in the community. Tablets were distributed in July 2017 to CHWs in Kandahar and Herat and at the end of August in Balkh. In Herat, only female CHWs implemented the intervention, while both male and female CHWs were trained to implement the intervention in Kandahar and Balkh. The rationale for including male CHWs from two provinces and excluding them in Herat was to explore whether male CHWs perceived value in and actually used the VL and whether gender-based issues affected tablet sharing and VL exposure when male CHWs were involved. In two provinces (Kandahar and Balkh), male CHWs were involved with an adaptive program design to engage men to view the HVL at health posts at activity inception; based on popular demand in Herat, this feature was added in October 2017.

### Study design

We conducted a mixed-methods, cross-sectional study to gauge exposure to and perception of the HVL in pilot communities. The assessment, conducted approximately 6 months after HVL introduction, comprised a household survey administered to target audiences through face-to-face interviews conducted by trained interviewers. In-depth interviews (IDIs) with CHWs, community health supervisors (CHS), and community leaders elicited opinions about operational feasibility, perceived HVL acceptability, and recommendations for HVL improvement. The study protocol was reviewed and approved by FHI 360’s Office of International Research Ethics (IRB #11659) and the MoPH Institutional Review Board (IRB #44010).

### Participants

For the household survey, married women^1^ who were currently pregnant or had at least one child 5 years of age or younger were eligible to participate, reflecting the HVL target population. Similarly, men who were married and had a child aged 5 years or younger were eligible to participate in the survey; male participants included husbands of the female participants and other men meeting these eligibility criteria in sampled households. We aimed to enroll 147 women and 147 men within each pilot district, a sample size sufficient to detect a 10% difference in exposure by sex between provinces with 80% power, assuming a 50% exposure rate, 5% non-response/decline rate, and a two-sided alpha of 0.05. We considered data among adult men and women from the formative work on which this intervention was designed and the 2013 Afghanistan Health Survey, where recalled CHW exposure in the last month was 29% and in the last 3 months was 45%, respectively [28]. The sample size was distributed equally across each village within pilot districts, then households were selected using a random walk technique [29]. Following household selection, the study was explained to male and female household members who met study eligibility criteria. Based on willingness to participate, one eligible woman or one eligible man was selected from each household.

For the IDIs, we recruited all CHWs implementing the HVL and CHS to probe their perceptions of client HVL acceptability. We also recruited community leaders, including representatives from *shura* and Family Health Action (FHA) groups. *Shuras* are community leadership councils typically comprising respected male village elders, while FHA groups are female-only groups present in some communities to provide support and information on health issues to local families. We chose to include these groups to further explore client impressions of the HVL, including ways to improve HVL access and appeal, for two reasons: [1] some members of these groups, particularly FHA groups, belong to the target HVL client population; and [2] collective community impressions about important events, such as HVL introduction, are communicated to leaders through their extensive social networks. The total number of interviews conducted, disaggregated by respondent type and district are presented in Table 1. We conducted IDIs with all consenting male and female CHWs in each province (except Herat where no male CHWs were formally trained at HVL inception), equivalent to up to 10 CHWs of each sex per province. One community leader from each village involved in the pilot activity was recruited for IDIs; purposive selection was used to ensure that five IDI participants in each district were *shura* members and at least three were FHA group members.

**Table 1:** Characteristics of IDI participants for VL feasibility assessment in Afghanistan, 2018

Participant group	Balkh		Herat		Kandahar		Total (by group)
	Male	Female	Male	Female	Male	Female	
Community Health Supervisor	2	0	2	0	2	0	6
Community Leader	5	3	4	0	5	5	22
Community Health Worker	5	9	4	10	10	9	47
Key Informant	0	0	0	4	0	1	5
**Subtotal** (by gender)	12	12	10	14	15	15	–
**Total** (by site)	24		24		32		80

### Measures

Separate household questionnaires were developed for men and women and pretested prior to data collection. While the questionnaires contained much of the same content, we surveyed men and women separately due to added questions around household decision-making dynamics and access to the HVL. These instruments included sociodemographic variables; questions about RMNCH knowledge, attitudes, and practices; and feasibility measures, including exposure to CHWs and the HVL (e.g. number and type of videos viewed). Acceptability measures included clients’ perceptions of whether video content was informative and culturally appropriate and whether respondents would recommend the HVL. We also asked participants’ opinions on how the HVL activity could be improved, including preferred HVL viewing sites and content recommendations.

Interview guides for IDIs focused on querying HVL implementation challenges, client acceptability, and operational issues. CHW IDI guides explored the following topics relevant to client impressions:

1. Experiences implementing the HVL
2. HVL features that CHWs or their supervisors liked or disliked
3. Challenges/barriers and facilitators to HVL use
4. Strategies to improve future HVL design and content

IDIs with community leaders and FHA members explored perceived community impressions of the HVL and ways to improve HVL access, appeal, content, and presentation format. Questions explored:

1. Perceptions of current and desired future HVL content
2. Perceptions of HVL implementation and acceptability within the community
3. Perceptions of CHW counseling and, more broadly, CHWs’ role in the community and perceived differences in HVL implementation by CHW sex.

### Data collection

Data were collected between February and April 2018. Trained, sex-matched staff introduced the study to household members, CHWs, CHS, and community leaders; verbal informed consent was obtained from eligible participants prior to conducting each survey and interview.

Completed paper questionnaires were checked in the field by the study team leader and an external monitor for quality assurance and then entered into an online database, with double-entry and reconciliation against all paper documents during central data cleaning. We descriptively analyzed quantitative data by province and sex with measures of central tendency and proportions using Stata version 13.1 (Stata Corporation, College Station, Texas, US). IDIs were audio-recorded, transcribed in Dari or Pashto, and translated to English. Qualitative data were coded and analyzed using NVivo 11 (QSR International, Doncaster, Victoria, Australia), based on a framework derived from key themes in the interview guides and emerging themes from transcripts. To ensure intercoder agreement, two trained analysts independently coded 10 transcripts (five CHWs, three CHS, and two community leaders) and discussed any discrepancies to ensure a common understanding of the codebook and interpretation of the text. As new codes were identified, the analysts reviewed previously-coded text and re-coded as necessary.

## Results

Findings from household surveys and IDIs regarding client HVL exposure and acceptability are presented together. Where possible—and following an overall description of participants—, we present quantitative and qualitative data thematically. The specific themes emerging from these data sources include HVL exposure and participation, receptivity to topic content, gender-related issues, and client recommendations for HVL improvement and expansion.

### Household survey respondent characteristics

Across the three pilot districts, 473 women and 468 men completed survey questionnaires. Sociodemographic characteristics of survey respondents are presented in Table 2. Female participants were generally younger (28 years vs. 36 years for men). Overall, the level of educational attainment was low, though more than twice as many men reported any formal education compared to women in all three provinces. At least half of male and female survey participants in all provinces reported having four or more children, with parity highest in Kandahar (Table 2). Most participants reported having electricity in their homes and a basic mobile phone, though other household items such as a television or computer were less common. Most men in Kandahar reported having a radio at home; however, radio access in Balkh and Herat was far less common.

**Table 2:** Sociodemographic characteristics of male and female post-intervention assessment participants in Balkh, Herat, and Kandahar provinces, Afghanistan 2018

Sociodemographic characteristic	Province % or mean (Standard Deviation (SD))					
	Women				Men	
	Balkh (*n* = 158)	Herat (*n* = 165)	Kandahar (*n* = 150)	Balkh (*n* = 158)	Herat (*n* = 161)	Kandahar (*n* = 149)
**Age** mean (+SD)	30.7 (+ 5.9)	26.8 (+ 6.5)	27.8 (+ 6.7)	34.9 (+ 8.6)	37.1 (+ 11.4)	36.9 (+ 10.6)
**Level of education**						
None	84.2	83.9	87.3	66.5	58.4	66.4
Primary (up to year 6)	8.9	6.0	4.0	12.7	31.1	8.7
Secondary (years 7–9)	0.6	6.6	0.7	4.4	4.4	7.4
High school, university, or vocational	3.2	3.6	4.0	8.2	6.2	9.4
Madrassah	3.2	0.0	4.0	8.2	0.0	8.1
**Number of living children**						
None (pregnant with first child)	1.9	5.4	2.7	4.4	2.5	1.3
1 child	10.1	12.5	8.7	11.4	9.9	11.4
2–3 children	26.0	32.7	30.0	31.7	24.2	23.5
4–6 children	49.4	37.5	38.7	38.6	40.4	36.9
More than 6 children	12.7	10.7	20.0	13.9	23.0	26.9
Declined to respond	0.0	1.2	0.0	0.0	0.0	0.0
**Number of years married**						
0–5 years	20.9	24.4	23.3	27.2	16.2	18.1
6–10 years	28.5	28.6	36.7	27.2	21.1	25.5
11–15 years	22.2	22.0	17.3	17.7	23.6	18.8
More than 15 years	26.6	12.5	22.0	27.9	37.9	37.6
Don’t know/declined to respond	1.9	12.5	0.7	0.0	1.2	0.0
**Household items**						
Electricity	91.8	66.7	76.0	98.7	91.3	81.2
Radio	11.4	7.1	42.7	12.7	21.1	69.1
Television	19.6	28.0	32.7	22.8	31.1	28.9
Mobile phone with Internet	4.4	13.7	18.7	8.2	15.5	18.1
Mobile phone without internet	69.6	68.5	88.7	91.8	90.1	88.6
Generator	3.8	2.4	18.7	4.4	6.2	15.4
Computer	0.6	1.8	11.3	0.6	2.5	5.4

We did not collect sociodemographic data for IDI participants (please refer to Table 1 for the number of interviews conducted, disaggregated by respondent type and district).

### HVL exposure

Overall, half or more of male and female survey participants reported CHW visits within the last month, predominantly at health posts and with some variation in exposure by province and sex (Table 3). Notably, 18% of women in Kandahar refused to say if they had seen a CHW. Nearly two-thirds of surveyed women in Balkh and Herat were aware of, and nearly as many had seen, at least one HVL video; however, relatively fewer women in Kandahar were aware of or reported exposure to the HVL (Table 3). HVL exposure among men in the survey sample was relatively low (less than 17%) across all provinces, although exposure among men in Kandahar exceeded that for women.

**Table 3:** Exposure of female and male participants to community health workers (CHWs) and associated CHW services in Balkh, Herat, and Kandahar provinces, Afghanistan 2018

CHW exposure indicator	Province (%)					
	Women			Men		
	Balkh (*n* = 158)	Herat (*n* = 168)	Kandahar (*n* = 150)	Balkh (*n* = 158)	Herat (*n* = 161)	Kandahar (*n* = 149)
***Any CHW exposure in the past month***	80.4	74.4	50.0	90.5	57.8	53.0
***Number of home visits by a CHW in last month***						
No visits	43.7	28.0	54.0	8.2	36.0	52.4
1 visit	19.6	22.0	10.0	36.7	9.9	9.4
2 visits	19.0	19.1	5.3	39.2	11.8	13.4
3 or more visits	15.2	21.4	10.7	5.1	18.0	8.1
Don’t know	2.5	8.9	2.0	10.1	21.7	8.7
Declined to respond	0.0	0.6	18.0	0.6	2.5	8.1
***Number of times visited a CHW at health post in last month***						
None	26.6	22.0	42.7	16.5	31.1	43.6
1 time	19.6	19.1	12.7	23.4	14.9	10.7
2 times	19.0	24.4	12.7	19.0	13.0	12.8
3 or more times	30.4	21.4	12.0	31.0	14.3	14.1
Don’t know	3.2	11.3	0.7	10.1	20.5	6.7
Declined to respond	1.3	1.8	19.3	0.0	6.2	12.1
***Heard of health video library*** ***program shown by CHWs***	63.3	67.3	31.3	37.8	23.0	28.2
***Respondent had seen at least one video shown by CHW***	60.1	67.3	15.3	8.2	11.2	16.8

A. **HVL access within communities**

To contextualize HVL exposure rates, we identified and explored themes surrounding barriers and facilitators of HVL access. CHW and community leader interviews suggested that communities underwent an adaptation period to the “new” HVL activity, which influenced the demand for and exposure to videos. Specifically, the novelty of the approach and technology was initially concerning to community members since women were not used to videos. Some CHWs reported community reluctance or resistance at HVL introduction, which was expressed using words like “strange” and “unnecessary,” or by relaying dissatisfaction or “bad thoughts” about the HVL.

*Some women think these videos are not necessary because their mothers in the past had not watched these videos and had no problem. I told them that facilities such as mobile phones, doctors, clinic and medicine were not available in the past. These facilities are for us to be informed and save ourselves from health problems.* (Female CHW, Herat)

These CHWs also said some community members raised concerns that HVL implementers were trying to “ruin our community” or change people’s religious beliefs, findings which contrast with reports from several interviewed community leaders that community members felt videos were “in line with their culture and Islam.” One community leader described outreach prior to HVL introduction with religious leaders to enlist their support as a means of facilitating HVL use.

*I invited all the CHWs that have tablets and arranged a large party where the religious leader was also invited. They watched all the videos and said that according to Islam and our area’s culture, the videos are fine. This was the reason that people don’t say anything negative about it.* (Male community leader, Kandahar)

Finally, a few CHWs also reported encountering resentment from clients reflecting a mistaken belief that CHWs were promoting HVL use within the community due to payment for HVL work. CHW-led discussions and viewing of the videos reportedly mitigated misperceptions and led to acceptance and satisfaction in most situations. For example, according to a male CHW in Kandahar, when CHWs encountered resistance, they would explain the reason for the HVL and its benefits to women and children or they showed videos to male family members or leaders to obtain approval.

B. **Topics viewed by community members**

Quantitative and qualitative data reflect preferences for certain video styles and topics as well as places for HVL viewing. With respect to the topics typically viewed by community members, we found that exclusive breastfeeding and PPH were the most commonly viewed video topics across all provinces, though exposure to specific topics varied by sex and province (Table 4). Moreover, while surveyed women tended to view videos during health post group sessions, surveyed men reported a variety of HVL exposure sites (Table 4). There were some differences between exposed men by HVL viewing site (own home was the most frequent in Kandahar compared to health post in Herat), topic (exclusive breastfeeding and LAM videos viewed more frequently in Herat), and video viewing by other household members (more frequently reported in Balkh). Irrespective of HVL viewing site and sex, more than 85% of survey respondents indicated that CHWs held group or individual counseling sessions following videos.

**Table 4:** Viewing characteristics of female and male participants who reported video library exposure in Balkh, Herat, and Kandahar provinces, Afghanistan 2018

	Women (***n*** = 231)			Men (***n*** = 56)		
	Balkh	Herat	Kandahar	Balkh	Herat	Kandahar
**Among those who saw any videos**	(*n* = 95)	(*n* = 113)	(*n* = 23)	(*n* = 13)	(*n* = 18)	(*n* = 25)
Health video topic seen:	%	%	%	%	%	%
Exclusive breastfeeding	51.6	57.5	69.6	30.8	63.2	26.9
Postpartum hemorrhage	35.8	68.1	47.8	69.2	31.6	3.9
Postpartum IUD	0.0	7.1	21.7	0.0	0.0	7.7
Lactational amenorrhea method	14.7	22.1	39.1	7.7	26.3	0.0
Women’s right to health care	1.1	9.7	52.2	7.7	15.8	19.2
Harms associated with early marriage	14.7	4.4	8.7	23.1	0.0	7.7
Chlorhexidine for cord care	15.8	22.1	34.8	7.7	0.0	0.0
Maternal tetanus vaccination	14.7	46.9	13.0	46.2	26.3	3.9
Child vaccination schedules	13.7	15.0	52.2	23.1	36.8	30.8
Exclusive breastfeeding/harms of bottle feeding	1.1	2.7	26.1	0.0	0.0	3.9
Early breastfeeding/colostrum	17.9	26.6	21.7	7.7	10.5	7.7
Other/nonspecific	6.3	3.5	26.1	0.0	15.8	26.9
Refused	1.1	0.9	0.0	0.0	5.3	19.2
**Place health video was viewed**						
Respondent’s home	15.8	26.6	30.4	23.1	36.8	38.5
Someone else’s home	27.4	8.9	26.1	38.5	5.3	26.9
At health post	52.6	53.1	34.8	38.5	47.4	26.9
Other	4.2	11.5	8.7	0.0	10.6	7.8
**Number of videos viewed**						
One	41.5	11.6	8.7	7.7	22.2	52.4
Two	38.3	33.0	17.4	69.2	33.3	33.3
Three or more	20.2	55.4	73.9	23.1	38.9	14.3
**CHW held discussion on topic after video was shown**	85.3	92.9	87.0	92.3	73.7	84.6
**Anyone else in household viewed video (among participants who viewed at least one CHW video)**	36.8	75.2	73.9	84.6	61.1	56.0

While CHWs described most video topics as being relatively easy to understand and learn, several IDI respondents reported noteworthy challenges. Specifically, older and uneducated clients initially had difficulty understanding videos, due to either content or a different local dialect. Illiteracy was also mentioned as contributing to difficulty understanding videos for a few women (mostly in Kandahar), though most CHWs remarked that the HVL was actually a solution to low literacy. Comprehension problems were reportedly readily resolved through post-video counseling or by watching the videos again.

*When we show the videos to women, we pause for a while and ask women what the video just said to make sure they understood correctly. If they did not understand well, we show the video again until they get the message. We ask those women who understand faster to explain to the rest of women …* (Female CHW, Herat)

Moreover, there were some specific HVL topics described as difficult for clients to understand. For example, CHWs in Herat considered the immunization video the most difficult to use, which required them to play the video multiple times. By contrast, CHWs in Balkh and Kandahar found the immunization video easy to discuss in counseling. A few CHWs said women did not understand the FP videos. One highlighted that people in their community like to have more than one child, perhaps indicating some resistance to FP discussion, irrespective of format.

### Acceptability of HVL

Surveyed women (*n* = 231) and men (*n* = 56) reporting HVL exposure generally welcomed the activity (Table 5). In addition, nearly all female (95.1–100%) and most male (81.1–92.9%) survey respondents across all provinces cited finding the post-video discussions useful. These high levels of HVL acceptance were also reflected in interviews with community leaders from all pilot communities, the majority of whom described the community’s feelings towards the HVL as “happy.”

A. **Feedback from women on HVL and video content**

**Table 5:** Video library program acceptability among women and men exposed to the video library in Balkh, Herat, and Kandahar provinces, Afghanistan, 2018

Acceptability indicator	Province (%)					
	Women			Men		
	Balkh (*n* = 95)	Herat (*n* = 113)	Kandahar (*n* = 23)	Balkh (*n* = 13)	Herat (*n* = 19)	Kandahar (*n* = 26)
**Liked getting health information from videos shown by CHWs on tablets**						
Very much	96.8	94.7	100.0	92.3	89.5	84.6
Somewhat	2.1	2.7	0.0	7.7	5.3	11.5
Not at all	1.1	0.0	0.0	0.0	0.0	0.0
Refused	0.0	2.7	0.0	0.0	5.3	3.8
**Most liked topics (among those who had viewed** **>** **1 video)**^a^						
	(*n* = 55)	(*n* = 99)	(*n* = 21)	(*n* = 12)	(*n* = 14)	(*n* = 10)
Liked all topics	40.0	35.4	9.5	0.0	21.4	10.0
Breastfeeding	10.9	19.2	23.8	50.0	21.4	10.0
Child vaccinations	18.2	12.1	23.8	16.7	21.4	20.0
Child health (general)	9.1	2.0	28.6	16.7	14.3	20.0
Pregnancy/care during pregnancy	7.3	21.2	9.5	16.7	14.3	0.0
Newborn care (e.g., chlorhexidine gel)	7.3	12.1	4.8	16.7	14.3	0.0
Birth spacing/FP	5.5	1.0	23.8	0.0	21.4	0.0
Birth/delivery (including PPH)	5.5	12.1	14.3	33.3	14.3	20.0
Handwashing/hygiene	0.0	5.1	9.5	0.0	0.0	0.0
Early marriage risks	3.6	1.0	0.0	0.0	0.0	0.0
Maternal health, including importance of access to health services	0.0	0.0	33.3	8.3	7.1	50.0
Does not remember/does not know	0.0	0.0	0.0	0.0	7.1	0.0
**Aspect respondent liked least about video(s)**^a^						
Did not dislike anything	78.9	66.4	65.2	76.9	73.7	65.4
FP/birth spacing topics or discussion	0.0	1.0	13.0	15.4	0.0	3.8
CHWs home/health post was crowded	0.0	1.0	0.0	0.0	0.0	0.0
Poor behavior of women in videos (offensive)	0.0	0.0	17.4	0.0	0.0	0.0
Respondent did not understand videos	1.1	0.0	0.0	0.0	0.0	0.0
Delivery at home	0.0	0.0	4.3	0.0	0.0	0.0
Early marriage	0.0	0.0	4.3	0.0	0.0	3.8
Don’t know/doesn’t remember	9.5	19.5	0.0	0.0	26.3	11.5
Other	0.0	0.0	8.7	7.7	0.0	3.8
No response	6.3	13.3	4.3	0.0	0.0	11.5
**Usefulness of post-video discussion**						
Very useful	95.1	100.0	100.0	91.7	92.9	81.8
Somewhat useful	4.9	0.0	0.0	8.3	7.1	18.2

^a^Percentages do not add to 100; multiple responses possible

Nearly all female survey respondents (94.7–100%) in the three provinces reported that they liked getting health information from videos shown by CHWs (Table 5). This finding was supported by qualitative data where CHWs described female clients’ reactions to the HVL as happy, eager, interested, and “having a good time together.” An FHA member from Balkh said that community members told her they “feel good” about the HVL as they now understand how and where to receive health care. She concluded, “They think their life is saved.” Several CHWs described situations where female clients or their husbands would call the CHW to conduct a home HVL session for visiting relatives and that “women would run” to the health post if new HVL content was available.

Breastfeeding, child vaccination, care during pregnancy, newborn care, and FP were among the video topics female survey respondents reported liking most (Table 5). Although IDI participants (CHWs and community leaders) reported that female clients appeared to like all content areas, responses from female survey participants suggest that preferences were more nuanced—40 and 35% of women who had seen more than one video in Balkh and Herat, respectively, reported liking all viewed videos, while only 10% of women in Kandahar cited liking all videos. When probed about the types of videos that were most appealing to beneficiaries, a few CHWs said clients especially enjoyed videos with humorous scripts or songs (e.g., the vaccine video). In addition, one CHW remarked that women liked topics that related directly to them.

When asked whether they disliked any HVL topics or other aspects, most female survey participants (65.2–78.9% across all provinces) indicated that there were no topics they disliked, though early marriage and FP topics were not liked by a small percentage of women (less than 3%) (Table 5). Despite women’s overall receptivity to the HVL topics, female survey participants and CHWs and community members identified some problematic aspects of the videos. In particular, four surveyed women in Kandahar remarked that “poor behavior” of women in a video they had watched was offensive; these participants had all seen the vaccination video and three of the four had seen the LAM video. Upon further inquiry, the complaint arose from perceptions that actors were immodestly dressed and covered. Moreover, in some HVL implementation areas, it is not culturally acceptable for women to be photographed or filmed, an issue which surfaced in a few interviews with CHWs. Specifically, some clients were reportedly concerned the tablet was being used to film HVL sessions. CHWs were explicitly told during training not to take pictures or videos of their clients, yet it seems some chose to document the sessions.

*Some women did not like filming them during the meeting even from the back of the meeting room. They either stepped aside or did not participate in the meetings any more. I told them that the ones who did not like to appear in the photo can stay aside so that she doesn’t appear in the film or picture.* (Female CHW, Herat)

B. **Feedback from men on HVL and video content**

Similar to their female counterparts, most surveyed men (84.6–92.3%) liked receiving health information from the HVL (Table 5). Although considerably fewer men than women were reached by the HVL, community leaders and CHWs perceived men to be generally pleased with and interested in the activity—this was evidenced by some men in Herat reportedly transferring videos to their mobile phones so they could watch and share them with peers. As perceived by one community leader, men’s interest in the HVL was driven by the desire to learn how to avoid illness-related expenses. Men in Kandahar were especially receptive to breastfeeding and maternal health (including importance of access to health services) topics, with 50% reporting liking these videos according to survey data.

When asked whether they disliked any HVL topics or other aspects, most male survey participants (65.4–76.9% across all provinces; Table 5) indicated that there were no topics they disliked, though FP topics and early marriage were not liked by a small percentage of men (less than 8%). This apparent discomfort with FP-related topics also emerged in interviews with CHWs: One Kandahar male CHW reporting that the condom method was the most difficult topic for men, who seemed embarrassed to hear about it. A few IDI respondents also reported gender-related concerns raised by community members, particularly men. A Balkh female CHW said “our men are sensitive to and do not accept gendered messages.” For example, a male CHW from Herat cited the video message of pregnant women needing to avoid strenuous work, saying that some people asked questions about this and disagreed with the video’s advice based on tradition and necessity. Moreover, though CHWs indicated that men frequently requested RMNCH topics for viewing, a few CHWs said men did not like content perceived to be only about women or about a specific topic.

*Men didn’t like the discussion about women...It was not interesting for men due to some points, such as pregnancy, but nobody was sad or hurt by the video library.* (Male CHW, Kandahar)

C. **Community desire to engage in future HVL activities**

Nearly all surveyed women and men exposed to the HVL reported desire to watch videos on other health topics in the future (Table 6). The most commonly requested topics included child health, particularly nutrition, and maternal health (without further specification). Many requested topics (e.g., breastfeeding, birth spacing, immunization) were already part of the HVL, though not all were available in both national languages. Men were more likely to request “general health” as a topic, while women tended to state specific future topics (Table 6). Respondents also suggested approaches for improving HVL use, with most inputs advising creating new videos, often for named specific topics; expanding HVL to other communities, particularly in remote areas; and providing more tablets in each community to expand reach.

**Table 6:** Women’s and men’s future interest in health videos among those who were exposed to the video library in Balkh, Herat, and Kandahar provinces, Afghanistan 2018

	Province (%)					
	Women			Men		
	Balkh (*n* = 95)	Herat (*n* = 113)	Kandahar (*n* = 23)	Balkh (*n* = 13)	Herat (*n* = 19)	Kandahar (*n* = 26)
**Level of interest in watching CHW videos on other health topics in the future**						
Very interested	95.8	93.8	95.7	92.3	89.5	84.6
Somewhat interested	1.1	2.7	4.4	7.7	0.0	15.4
Not at all interested	1.1	0.0	0.0	0.0	0.0	0.0
Refused to answer	2.1	3.5	0.0	0.0	10.5	0.0
**Other requested health topics participant would like to see in videos**^a^						
Child health/nutrition	36.8	14.2	43.5	23.1	21.1	26.9
Maternal health	18.9	8.0	34.8	15.4	0.0	26.9
General health	9.5	4.4	21.7	23.1	21.1	26.9
Pregnancy/care during pregnancy	7.4	8.0	13.0	0.0	0.0	0.0
Birth spacing/FP methods	3.2	10.6	8.7	7.7	0.0	3.8
Hygiene and safe drinking water	2.1	6.2	13.0	0.0	0.0	0.0
Vaccinations for children and mothers	15.8	6.2	4.3	7.7	0.0	11.5
Delivery/care during delivery	2.1	3.5	8.7	0.0	0.0	0.0
Mental health	0.0	0.0	0.0	0.0	0.0	11.5
Breastfeeding/postnatal care	2.1	8.0	4.3	0.0	0.0	0.0
Other	2.1	6.2	4.3	0.0	0.0	0.0
Don’t know/no specific suggestion	15.8	47.8	4.3	0.0	0.0	26.9
No response	0.0	5.3	0.0	0.0	0.0	15.4

^a^Percentages do not add to 100, multiple responses possible

Congruent with high proportions of community members desiring further HVL programming, CHWs and community leaders echoed the perceived desire and need for continued HVL programming. Importantly, the HVL reportedly increased acceptance of CHWs’ role in the community.

*Women were thankful to watch these videos. Previously, women did not trust in us* [CHWs] *that much but after watching videos, they trust more in us because they have watched and liked all topics*. (Female CHW, Herat)

All community leaders recommended that the HVL be sustained and extended to other communities. A male health *shura* member expressed a desire for many to reap HVL’s benefits:

*The head of Khoshab village was talking with me on the phone last night. I asked him about his idea about this health program and told him this is the best program for Afghan people and have benefits in this world … I suggest you to scale up this program that most of the people get its advantages.* (Male community leader, Kandahar)

## Discussion

In this analysis, we examined the feasibility and acceptability of using videos to enhance health education sessions delivered to community members by CHWs from the client perspective. Results from this assessment are mixed. While men and women exposed to the HVL activity overwhelmingly approved of the approach, the HVL pilot experienced varying degrees of challenges reaching intended target populations. Relatively low reported HVL exposure among women in Kandahar and among men in all provinces indicates a need for activity design modification. Specifically, broad community notification and endorsement by community leaders at HVL introduction and ongoing engagement with these stakeholders is critical to ensuring that community members, particularly women, are able to access health posts for HVL exposure and subsequent care and referral needs. Other notable findings were the initial resistance to HVL use within communities, disproportionate reported client exposure to a few video topics, and client recommendations to improve HVL activity through expanded content and geographic coverage.

Regarding feasibility, HVL exposure among female target audiences in Herat and Balkh districts was nearly as high as reported CHW engagement in the last month. HVL may have driven some of this engagement, as reported rates of CHW exposure in the 2015 Demographic and Health Survey and other literature are lower than those reported in these districts in the month following HVL introduction [2, 11, 12]. In Kandahar, female target audiences had both lower CHW and HVL exposure, findings likely attributable to two factors. First, there are significant cultural limitations to women’s movement outside the household; male family members (*mahram*) must accompany the woman to all places [30]. Indeed, that nearly 20% of women in Kandahar refused to say whether they had seen a CHW reflects the culturally conservative nature of the province—we speculate that given the negative community perceptions regarding women leaving the home (e.g. to see a CHW at a health post), female participants from Kandahar were not inclined to disclose whether they had seen a CHW in the past month. These cultural constraints also apply to female CHWs, whose ability to conduct health post and home visit sessions was limited compared to their Balkh and Herat counterparts. In this study but reported elsewhere, female CHWs reported infrequent or forfeited tablet possession as tablets were ceded to male CHWs due to gender norms regarding property ownership and perceived need (personal communication, L. Lorenzetti), as noted in another study of Afghan CHWs [11]. Social norms resulting in male CHW tablet possession may explain why men in Kandahar were more likely to have viewed HVL videos than women. With respect to low HVL exposure among men, pilot implementation did not include specific male engagement events, likely contributing to low male target audience exposure across districts.

The HVL was widely viewed as acceptable; however, CHWs described initial resistance from some community members due either to cultural concerns over video content where humans were depicted or the HVL’s orientation toward female clients and perceived lack of effort to include male clients. Based on other formative research in Afghanistan, rural communities like those in this pilot have limited television exposure [3]; thus, we surmise that this new visual media source may have required time to gain credibility and acceptability. Men are the gatekeepers for health care access, with degree varying by region [2]. We believe the lack of male engagement with HVL introduction, particularly in Kandahar, initially limited both male and female HVL exposure but may have improved with time as men became more familiar with and recognized the value of the HVL. The broader HVL coverage among male clients in Kandahar may also have been positive in the sense that it reflects eventual male acceptance of HVL and paves the way for eventual greater female access. To establish a more conducive environment for HVL uptake in scale-up, we have added an introductory HVL session (“soft opening”) with key influencers that men trust (i.e. community *shura,* FHA groups, and community mullah/imam) *prior* to community introduction. These “soft openings” entail trained CHWs introducing the program, showing leaders the videos and having them experience the counseling, and providing a brief overview of the pilot assessment’s findings. Other activity steps to engage men in HVL during scale-up include the provision of a separate tablet to male CHWs, and the scheduling of special male audience times, such as after Friday prayers.

Overall, among exposed audiences, most clients strongly agreed that they would like to receive health messages via the HVL in the future. As with other video interventions to improve RMNCH knowledge and service uptake in low-resource settings [22–24, 31], IDI data indicated that the HVL visual presentation format augmented CHW counseling and made concepts easier for clients to understand. Moreover, our findings that clients and their families sought HVL session attendance and that HVL elevated community perceptions of CHW professionalism are also consistent with results from similar interventions [21, 23, 24]. That many participants, specifically community leaders, in our study spontaneously recommended scale-up to other communities speaks to the HVL’s acceptability in pilot districts.

Though viewed content was deemed largely acceptable, some topics were reportedly viewed seldom or not at all by exposed clients. CHWs may have curated videos based on social norms and topic sensitivity, reflected by relatively lower viewing of FP-specific videos. We believe that CHWs know by experience and through their national CHW training that counseling and engagement around sensitive topics like FP are best done in individual sessions. The HVL tended to be used in group settings, which may have precluded FP discussions and thus resulted in low demand for these videos. However, it is unclear whether lower viewing levels were due to CHWs’ reservations about the content, low client demand, or position of the FP video folder toward the end of the list on the tablet. The training guide did not mandate an order to showing the videos but CHWs were encouraged to show up to three videos of different topics at each session over several weeks until all videos had been viewed. We purposefully chose pilot sites that varied geographically and culturally across Afghanistan, yet films depicting women’s faces or dressing in a style from another part of the country were reportedly jarring to some female clients in Kandahar. The HVL included videos in both Dari and Pashto; however, showing Dari videos dubbed in Pashto rather than re-filming them with regionally normative changes in dress and mannerisms possibly prompted this reaction.

Clients provided recommendations to improve the HVL. Suggestions mainly centered on creating new video content and expanding HVL to other communities. Other priority recommendations included ensuring that HVL message content is clear and simple, as the immunization and preventing early marriage videos in particular were difficult to understand for some audiences. Participants also recommended that all videos be available in the language of the viewing district and that all CHWs in the community be given tablets.

Several limitations should be considered when interpreting these data. First, the study relied on convenience sampling for both the survey and IDIs; thus, findings cannot be generalized beyond the study samples. A second limitation is that the study enrolled substantial numbers of participants who were not exposed to the HVL, further limiting our ability to report participant experiences. That lower proportions of women in Kandahar and men overall reported HVL exposure is difficult to interpret due to the non-probability nature of our sample and small numbers. The extent to which coverage in these areas was lower or that our sample simply missed those who were exposed is unknown. We also don’t know to what extent the opinions of those study participants who were exposed to the HVL reflect broader community sentiment with regard to HVL feasibility or acceptability. Last, all findings are based on self-report from study participants; we did not attempt to verify reported exposure to specific videos.

## Conclusions

We conclude that using the tablet-based HVL to enhance CHW-led RMNCH education was generally feasible and—among exposed women and men—acceptable in culturally-diverse settings in Afghanistan. Digital tools such as the HVL are not a panacea for increasing RMNCH knowledge and service utilization in this and other under-resourced settings; however, provided sustained engagement with CHWs, community leaders, and other key stakeholders (e.g. MoPH), these tools offer a promising, additional channel through which to communicate important RMNCH messages. In response to these assessment findings, the project has modified the HVL intervention to include the following: development of new video content, particularly for high-impact RMNCH interventions, nutrition, and FP; holding a “soft opening” for community leaders and key influencers prior to HVL introduction to the community at large; and pretesting all videos across a diverse range of settings prior to HVL inclusion. Based on these pilot and other programmatic results, we have added a line of videos called Opinion Leader videos, where CHWs identify community members who have successfully adopted a new behavior and navigated barriers to doing so, such as facility-based delivery and are willing to be interviewed and filmed. In the videos, these community members describing how they were able to adopt the new behavior and, by doing so, incite conversation around and solutions to transform harmful social norms.

As this assessment was not designed to measure the effect of HVL on RMNCH-related knowledge or behaviors, we recommend a rigorously designed evaluation to measure the HVL’s effect on knowledge or on targeted health-related behavioral outcomes prior to implementation at large scale. Further research is also warranted to explore whether this tablet-based tool could be culturally-adapted for RMNCH promotion efforts in other conflict-affected settings.

## Data Availability

The de-identified quantitative datasets from the study are available at https://dataverse.harvard.edu/dataset.xhtml?persistentId=doi:10.7910/DVN/YAGDV0. Qualitative datasets are available from the corresponding author on reasonable request.

## Abbreviations

ASHA: Accredited Social Health Activist
BPHS: Basic Package of Health Services
CHW: Community health worker
CHS: Community health supervisor
FHA: Family Health Action
FP: Family planning
HPD: Health Promotions Department
HEMAYAT: Helping Mothers and Children Thrive in Afghanistan
HVL: Health video library
IDI: In-depth interview
IRB: Institutional Review Board
IVR: Interactive voice response
LAM: Lactational Amenorrhea Method
MoPH: Ministry of Public Health
PPH: Postpartum hemorrhage
PPIUCD: Postpartum intrauterine contraceptive device
RMNCH: Reproductive, Maternal, Newborn, and Child Health
SMS: Short message service
USAID: United States Agency for International Development

## Footnotes

1 Married individuals are considered adults in Afghan society; thus, no age range was added to the eligibility criteria.
